# Grouping strategies for assessing and managing persistent and mobile substances

**DOI:** 10.1186/s12302-024-00919-4

**Published:** 2024-05-21

**Authors:** Parviel Chirsir, Emma H. Palm, Sivani Baskaran, Emma L. Schymanski, Zhanyun Wang, Raoul Wolf, Sarah E. Hale, Hans Peter H. Arp

**Affiliations:** 1 Luxembourg Centre for Systems Biomedicine (LCSB), University of Luxembourg, 6 Avenue du Swing, 4367 Belvaux, Luxembourg; 2 Department of Environmental Engineering, Norwegian Geotechnical Institute, 0806 Oslo, Norway; 3 Technology and Society Laboratory, Empa-Swiss Federal Laboratories for Materials Science and Technology, 9014 St. Gallen, Switzerland; 4 TZW: DVGW-Technologiezentrum Wasser (German Water Centre), Karlsruher Straße 84, 76139 Karlsruhe, Germany; 5 Department of Chemistry, Norwegian University of Science and Technology (NTNU), 7491 Trondheim, Norway

**Keywords:** PMT, vPvM, Transformation products, Hazardous properties, Regrettable substitution, Substance grouping, Chemical regulation, Persistent, Mobile, Toxic

## Abstract

**Background:**

Persistent, mobile and toxic (PMT), or very persistent and very mobile (vPvM) substances are a wide class of chemicals that are recalcitrant to degradation, easily transported, and potentially harmful to humans and the environment. Due to their persistence and mobility, these substances are often widespread in the environment once emitted, particularly in water resources, causing increased challenges during water treatment processes. Some PMT/vPvM substances such as GenX and perfluorobutane sulfonic acid have been identified as substances of very high concern (SVHCs) under the European Registration, Evaluation, Authorisation and Restriction of Chemicals (REACH) regulation. With hundreds to thousands of potential PMT/vPvM substances yet to be assessed and managed, effective and efficient approaches that avoid a case-by-case assessment and prevent regrettable substitution are necessary to achieve the European Union's zero-pollution goal for a non-toxic environment by 2050.

**Main:**

Substance grouping has helped global regulation of some highly hazardous chemicals, e.g., through the Montreal Protocol and the Stockholm Convention. This article explores the potential of grouping strategies for identifying, assessing and managing PMT/vPvM substances. The aim is to facilitate early identification of lesser-known or new substances that potentially meet PMT/vPvM criteria, prompt additional testing, avoid regrettable use or substitution, and integrate into existing risk management strategies. Thus, this article provides an overview of PMT/vPvM substances and reviews the definition of PMT/vPvM criteria and various lists of PMT/vPvM substances available. It covers the current definition of groups, compares the use of substance grouping for hazard assessment and regulation, and discusses the advantages and disadvantages of grouping substances for regulation. The article then explores strategies for grouping PMT/vPvM substances, including read-across, structural similarity and commonly retained moieties, as well as the potential application of these strategies using cheminformatics to predict P, M and T properties for selected examples.

**Conclusions:**

Effective substance grouping can accelerate the assessment and management of PMT/vPvM substances, especially for substances that lack information. Advances to read-across methods and cheminformatics tools are needed to support efficient and effective chemical management, preventing broad entry of hazardous chemicals into the global market and favouring safer and more sustainable alternatives.

## Background

In 2019 water pollution was estimated to cause 1.4 million premature deaths globally [1]. Improving water quality by reducing pollution is also defined as one of the tasks in the Sustainable Development Goals [2]. Meanwhile, the number of known chemicals that are in use is increasing dramatically. Over 350,000 chemicals and mixtures have been registered in the global market over the past 50 years [3], while the largest chemical databases contain over 100 million chemicals, with PubChem [4] and the Chemical Abstracts Service (CAS) registry containing 116 million [5] and 219 million [6] chemicals, respectively, as of January 2023. Chemical production and pollution are outpacing global assessment capacity, posing more risks to human health, wildlife, and the environment [7, 8]. In 2021, about 222.8 and 85.3 million tonnes of chemicals hazardous to human health and the environment, respectively, were consumed in the European Union (EU) [9]. Some of these chemicals are persistent, mobile and toxic (PMT) substances or very persistent and very mobile (vPvM) substances, collectively referred to as PMT/vPvM substances.

**Fig. 1:**
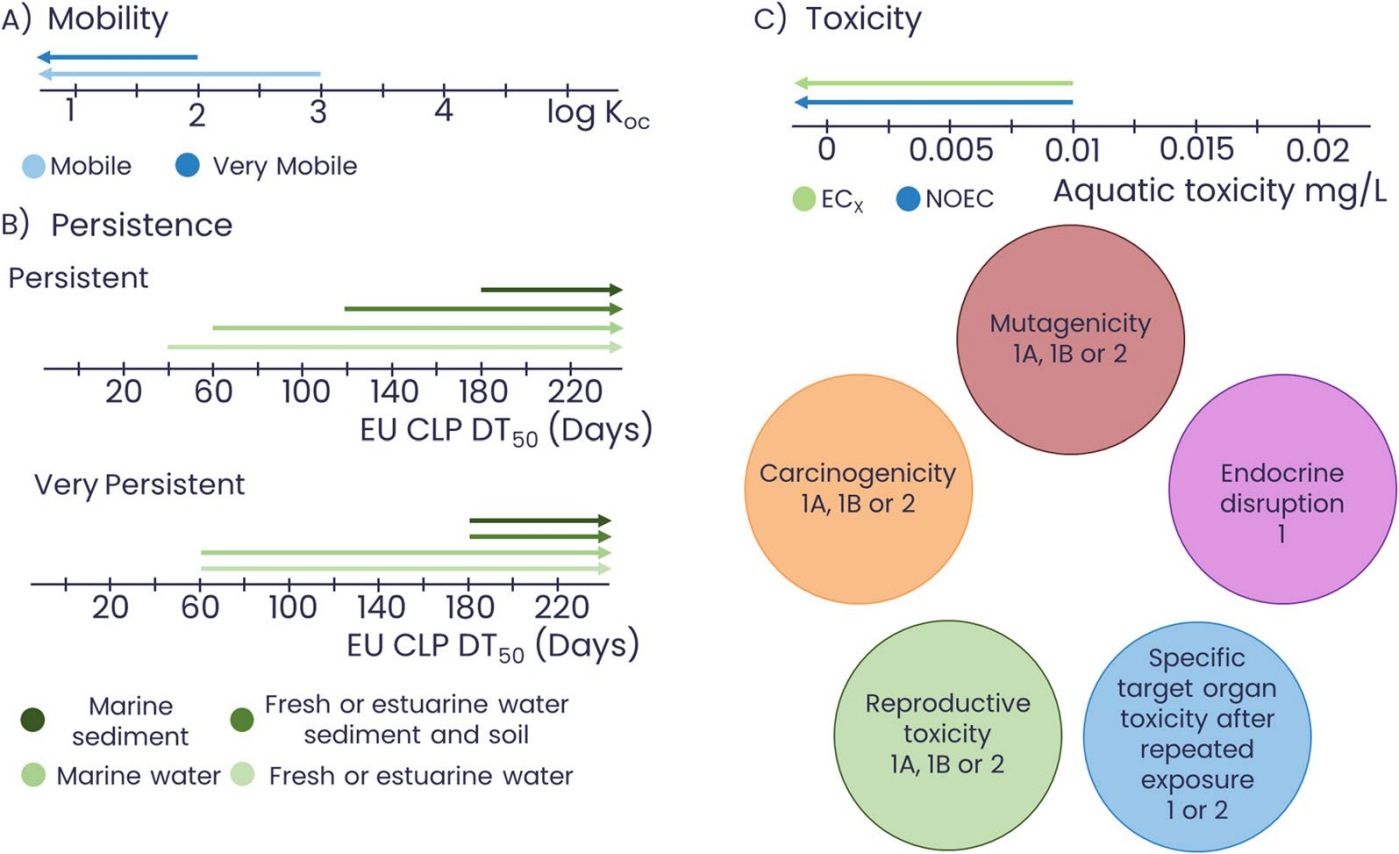
EU CLP criteria [11] for PMT/vPvM substances. **A** Mobility criteria based on log K_OC_ (blue). **B** Persistence criteria, with persistent in green and (**C**) toxicity criteria, including carcinogenicity, mutagenicity, endocrine disruption, specific target toxicity and reproductive toxicity (1A, 1B, 1 and 2 refer to the categories for that criterion) as well as concentrations, where long-term no observable effect concentration (NOEC), and effect concentration (EC_x_) for marine and freshwater organisms refers to the concentration where a toxic effect was observed in a given percentage of the population, e.g., 10% for EC_10_ at less than 0.01 mg/L

PMT/vPvM substances have been listed by the European Commission’s Scientific Committee on Health, Environmental and Emerging Risks (SCHEER) as one of the 14 emerging problems that could impact human health or the environment [10]. Recently, the EU Chemicals, Labelling and Packaging (CLP) regulation (1272/2008) introduced the new hazard classes PMT and vPvM, as well as defined criteria for PMT/vPvM substances based on chemical properties [11], as presented in Fig. 1. These substances do not degrade in the environment over an appreciable timescale and are easily transported through water and aquatic ecosystems due to poor sorption to soil and sediments [12–14]. PMT/vPvM substances can be found in a wide range of applications and sources, including in industrial processes, fire-fighting foams, and consumer products, such as food, cosmetics and furniture [15]. They can cause long-term harm to humans and the environment and are also costly and difficult to remove from drinking water [16–19]. PMT/vPvM substances have been suggested to have an equivalent level of concern as persistent, bioaccumulative and toxic (PBT) substances or very persistent and very bioaccumulative (vPvB) substances. GenX and perfluorobutane sulfonic acid (PFBS), both classified as PMT/vPvM substances by the German Environmental Agency (UBA) [20] (but not yet officially classified under the CLP Regulation), have been identified as substances of very high concern (SVHCs) under the European Registration, Evaluation, Authorisation and Restriction of Chemicals, REACH, regulation (EC 1907/2006) [12]. The commonality between PMT/vPvM and PBT/vPvB substances is their elevated potential for long-lasting and long-range exposure when released to the environment in substantial quantities. Their difference is related to the exposure pathway, with PMT/vPvM substances likely to be first monitored in water resources, where they are most likely to accumulate and PBT/vPvB substances being first monitored in the food supply, humans and living organisms. Though this was not stated within the CLP regulation [11], it may be assumed that these hazard classes were also introduced due to concerns from enhanced exposure potential leading to unforeseen effects, be they odour, aesthetic, geophysical, economical or other than established toxicological criteria. As an example, REACH Annex 1, Sect. 0.10 refers to “particular effects, such as ozone depletion, photochemical ozone creation potential, strong odour and tainting, for which […] the risks associated with such effects shall be assessed on a case-by-case basis”. Considering these concerns, a key consideration for vP substances is to further check if they exhibit enhanced exposure-related properties (vB or vM), toxicological or other hazards, following the current regulatory approaches.

Furthermore, due to the high mobility of PMT/vPvM substances, many of them can break through artificial barriers in wastewater treatment plants, including granular activated carbon filtration and ultrafiltration systems, posing challenges for removal and remediation [12, 21]. These substances can be further transported through natural media, such as soils, riverbanks, aquifers and groundwater, making them hard to contain and remove from the environment. As such, these substances are problematic in drinking water, and many are detected frequently in European surface waters [12]. For example, 1,4-dioxin was found in Bavarian surface waters [22] and melamine in the Netherlands, France and Belgium [23].

Approximately 2% of identifiable unique chemicals registered under REACH are considered to meet the PMT/vPvM criteria (259 out of 13,405 unique, identifiable chemicals registered under REACH); however, the true number is likely higher due to data gaps related to persistent, mobility or toxicity, with a potential maximum of 28% of REACH registered substances (3677 out of 13,405 substances) [24]. Developing and applying substance grouping strategies is one way to potentially identify and manage PMT/vPvM substances more effectively. There are two main motivations for a substance grouping approach. The first is to expedite hazard assessments related to the large number of substances being introduced to the global chemical market. The second is to avoid regrettable substitution caused by drop-in substitution [25], where one substance is replaced by another with similar hazardous properties and effects [26]. Thus, this paper aims to explore how grouping strategies can be generally developed and used to identify, assess and manage new, emerging and well-known PMT/vPvM substances. To provide context, the paper first provides an overview of PMT/vPvM substances, including scoping the numbers of substances covered by existing definitions, reviewing previous successful grouping strategies, and determining the relevance of grouping strategies in the context of PMT/vPvM substances while exploring the future efforts required to achieve this effectively. The principle aim of this article is not specifically targeting the ECHA technical guidance for PMT/vPvM substance assessment, but rather it is on how grouping strategies can be used to screen, assess, regulate and manage groups of PMT/vPvM substances more efficiently.

## Methods

### Definition of key terms

The International Union of Pure and Applied Chemistry (IUPAC) defines a chemical substance as “*matter of constant composition best characterized by the entities (molecules, formula, units, atoms) it is composed of. Physical properties such as density, refractive index, electric conductivity, melting point *etc*. characterize the chemical substance*” [27]. The term “substance” in this article is used in this context. Databases also refer to chemical entities as compounds and substances, but the context may be different. PubChem, for instance, define these as “*a substance is a chemical sample description provided by a single source and a compound is a normalized chemical structure representation found in one or more contributed substances*” [28]. Since this article later refers to calculations performed on PubChem queries, “compound” in this article refers to a chemical that fulfils the definition of a compound according to PubChem with a unique PubChem Compound Identifier (CID). A mixture, according to IUPAC, is a “*portion of matter consisting of two or more chemical substances called constituents*” [29]. Mixtures can be simple (e.g., xylene is a mixture of three isomers, *o*-xylene, *m*-xylene and *p*-xylene) or complex (e.g., C_9_–C_14_ alcohols, or mineral oils). The latter are often referred to as “substances of Unknown or Variable composition, Complex reaction products or Biological origin” (UVCBs) [30].

A group of substances is defined by REACH as substances that have “(1) a common functional group; (2) the common precursors and/or the likelihood of common breakdown products via physical and biological processes, which result in structurally similar chemicals; or (3) a constant pattern in the changing of the potency of the properties across the category.” (REACH Regulation EC No 1907/2006, Annex XI, Sect. 1.5) [31]. In other words, the term “group” and “category” are used interchangeably in this article. As an example, two widely regulated groups of substances that fulfil all three aspects of grouping substances in the REACH regulation are dioxins (polychlorinated dibenzo-*p*-dioxins and polychlorinated dibenzo-furans) [32] and polychlorinated biphenyls (PCBs) [33], which are composed of about 210 [34] and 209 individual congeners, respectively [35].

### Literature analysis

To obtain relevant literature to underpin the arguments in this paper, a comprehensive literature search spanning both policy-related documents (e.g., regulations, guidelines, analysis, dossiers) and scientific literature was conducted using keywords like "persistent, mobile and toxic", “very persistent very mobile”, "substance grouping" and "chemical regulation" in electronic databases, such as PubMed and Google Scholar, while filtering for relevance. As the study focus area was mainly the European Union, recent policy documents in the EU and United Nations were considered most relevant due to the greater activity on PMT/vPvM substances, while detailed policy analysis of areas outside the European Union was considered out of scope. Scientific studies focused on grouping strategies for the assessment and management of substances, including chemicals, pollutants, and contaminants that exhibit persistence and mobility in the environment were also considered relevant. Backward citation searching was also conducted from the list of relevant articles to identify additional studies that may have been overlooked during the electronic search. By systematically executing the search strategy across multiple databases and supplementing it with hand-searching reference lists, a comprehensive selection of relevant literature with findings focused on the topic was compiled and considered in this article.

### Cheminformatics analysis

For cheminformatics analysis, the search functions available in the PubChem database [4] were used to obtain the structural and substructural data of compounds of interest. The OPERA ReadyBiodegradable model version 2.9 [36] was used to predict the persistence of compounds, along with the KOCWIN model version 2.00 [37] to predict the mobility (log *K*_oc_ values) of the compounds from the PubChemLite for Exposomics data set, Version 1.27.0 [38] and other subsets of compounds extracted from PubChem. Further details of other data sets used can be found in the "availability of data and material" section. The toxicity predictions were performed with MS2Tox version 0.3.2 [39]. Data processing was performed in R version 4.3.1 [40] using the packages Tidyverse version 2.0.0 [41, 42] and ggplot2 version 3.4.3 [43, 44] for data transformation and plotting, and RChemMass 0.1.28 [45] for obtaining the exact mass before MS2Tox calculations. Additional information is given in the “Cheminformatics challenges in grouping PMT/vPvM substances” section.

## Overview of PMT/vPvM substances

Assessment of the number of substances being introduced to the global chemical market that may be PMT/vPvM substances requires a clear set of criteria and the ability to scale the application of these criteria to large numbers. The EU Chemicals, Labelling and Packaging (CLP) regulation (EC 1272/2008) criteria for PMT/vPvM substances [11] are displayed in Fig. 1. The *mobility* (M) aspect (see Fig. 1A) is defined based on the organic–carbon–water partition coefficient (*K*_OC_, with units of L/kg) of a chemical which is then log transformed to log *K*_OC_ values, this is used as a proxy to describe the sorption potential of a chemical to organic carbon. Since experimental log *K*_OC_ data are rare, it was suggested that the logarithmic octanol–water partition coefficient (log *K*_OW_ or log *P*) or the pH-adjusted log *D*_OW_ (or log *D*) could be used as a screening parameter for hydrophobicity, where high-quality log *K*_OC_ data are not available [24, 46]. Though the two parameters have some variance, particularly for ionogenic substances, substances with very low log *D*_OW_ values tend to have very low log *K*_OC_ values, hence, log *D*_OW_ values were seen as a good alternative screening parameter for mobility. As such they are often used in a complementary fashion (see [24, 47] for more details), where log *K*_OW_ and log *D*_OW_ data are used when log *K*_OC_ data are unavailable. *Persistence* (P) is defined based on the half-lives of a chemical in different environmental water, sediment and soil systems (see Fig. 1B), and toxicity (T) is defined based on one or a combination of the following: carcinogenicity, mutagenicity, reproductive toxicity, endocrine disruption and specific target organ toxicity after repeated exposure as well as long-term no-observed effect concentration (NOEC) and effect concentration at a given percentage of the population (EC_x_) shown in Fig. 1C.

**Fig. 2:**
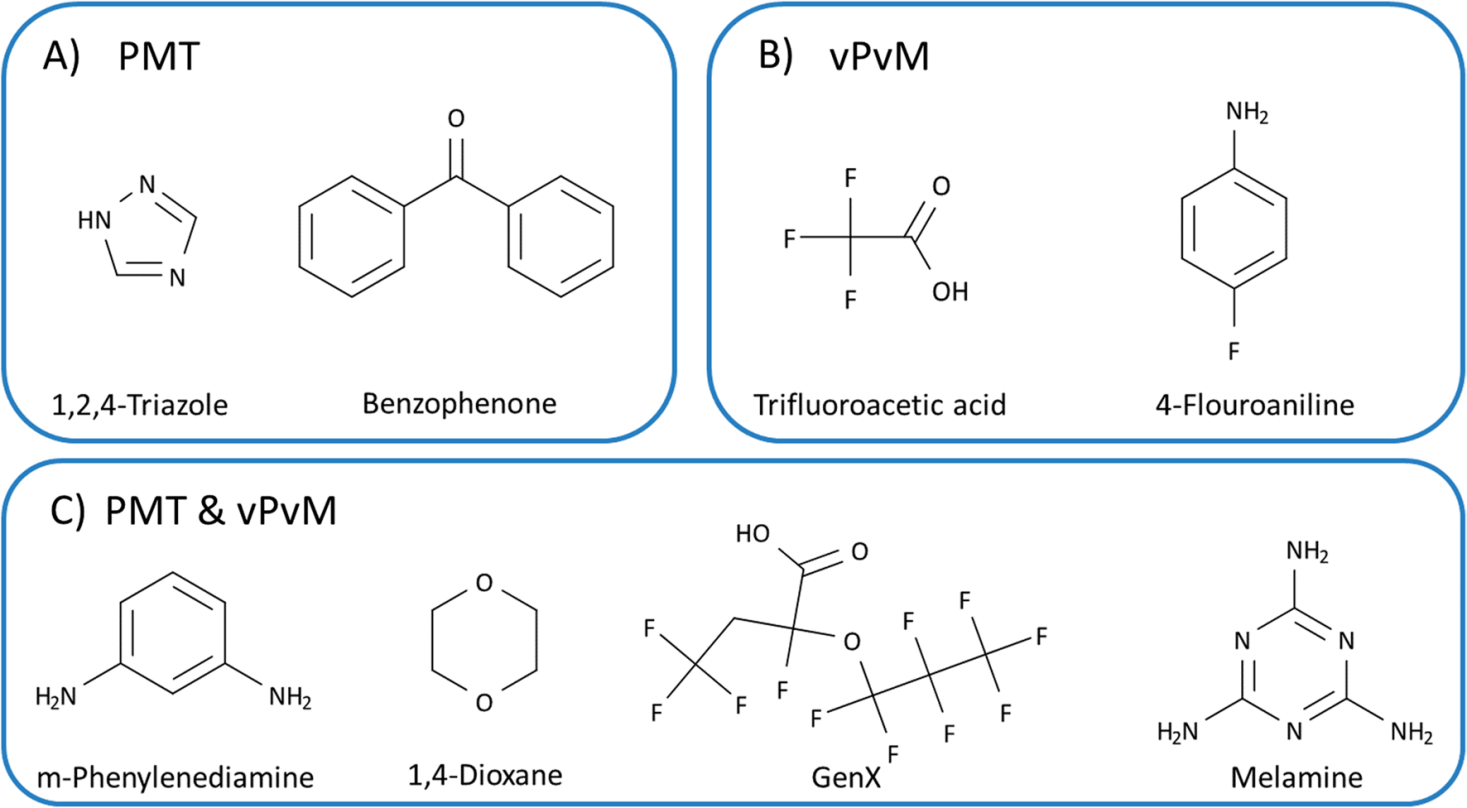
Selected examples of potential PMT/vPvM substances from the UBAPMT list assessed by Arp and Hale (2022) as meeting the criteria [20, 24]. **A** PMT substances; **B** vPvM substances; **C** both PMT and vPvM substances

A compound is a considered a PMT substances when it fulfils the persistence, mobility and toxicity criteria (i.e., a combination of at least one persistency criterion, one mobility criterion and one toxicity criterion are met) or in the case of vPvM at least one of the criteria for very persistent and the very mobile criterion must be met (see Fig. 1). Some examples of PMT/vPvM substances are shown in Fig. 2.

There are several published lists containing PMT/vPvM substances (e.g., the PMT list by Holmberg et al., UBAPMT, EAWAGPMT, UFZHSFPMT, ZEROPM and PMTPFAS) [20, 48–52]. The later five have been digitized and are hosted on the NORMAN Suspect List Exchange (NORMAN-SLE) [53, 54] and thus openly available with a CC-BY license. The UBAPMT suspect list contains substances currently registered under REACH that meet the proposed PMT/vPvM criteria set by UBA in 2019 [47]. The original list has 254 substances [55, 56], while the 2022 revised version has 340 substances [57, 58] due to an increase in chemicals registered in REACH and updated chemical information [20]. The EAWAGPMT list contains 1,156 compounds identified in groundwater by Kiefer et al. [49, 59]. This list contains a mix of compounds on other PMT lists as well as compounds on the Swedish Chemical Agency (KEMI) market list [60] that have a low log *D*ow and high water exposure index, while the UFZHSFPMT list includes 1,063 potential persistent mobile compounds detected in surface water as described by Neuwald et al. [50, 61, 62]. The ZEROPMBOX1 contains 38 compounds, including representative per- and poly-fluoroalkyl substances (PFAS), triazines and triazoles used to start the H2020 ZeroPM project [51]. Finally, the PMTPFAS list contains 180 fluorinated compounds extracted from the UBAPMT, EAWAGPMT and UFZHSFPMT lists [52]. In combination, the five lists contain 2,081 unique compounds, but this does not represent all PMT/vPvM substances in the global market.

## Grouping and regulations

Assessing and regulating individual PMT/vPvM substances that fall within a PMT/vPvM substance group is inefficient and more time-consuming than assessing an entire group. Past strategies have led to drop-in replacements, subsequently referred to as regrettable substitution [25]. Assessing chemicals individually also ignores cumulative exposures and risks of groups of substances [63]. Considering primarily the structural similarity and similar properties of PMT/vPvM substances, it may be more feasible and prudent to regulate these substances as a group. The idea of substance grouping based on the relationship between hazard and structural similarity is not new, since many of the very first organic substances to be regulated were groups sharing a similar structure. The successes of regulation in managing substances as groups, such as ozone-depleting substances (ODS) under the Montreal Protocol [64, 65] and specific groups of persistent organic pollutants (POPs) under the Stockholm Convention [66] are discussed below. Moreover, successfully grouping substances for regulation not only accelerates but also prevents and reduces inconsistencies in the regulatory process [67]. Grouping substances can enhance chemical safety management, facilitating the identification of regulated substances and potential substitutes for harmful ones [67].

While there may be several legislations about grouping substances globally, this section focuses on the use of grouping in EU legislation. In the EU, the European Chemical Agency (ECHA) coordinates the REACH regulation for the restriction, evaluation and authorization of substances based on hazard classifications and the CLP regulation for labelling based on hazard classifications. REACH Annex XI Sect. 1.5 specifies how groups or categories of substances can be defined and which regulatory actions can be applied [31]. It states that “*substances whose physicochemical, toxicological and ecotoxicological properties are likely to be similar or follow a regular pattern as a result of structural similarity may be considered as a group, or ‘category’ of substances*” [31]. The toxicological or hazard properties referred to are described in the CLP regulation shown in Fig. 1.

According to Article 36 of the CLP regulation, certain substances, including those with respiratory sensitizing properties (category 1), germ cell mutagenicity, carcinogenicity, and reproductive toxicity (categories 1A, 1B and 2), are considered hazardous and are subject to harmonized classification. This also applies to active substances listed in Regulation (EC) No 1107/2009 and Regulation (EU) No 528/2012 [68, 69]. Moreover, Article 57 of REACH includes substances that are hazardous to the environment and are also subject to harmonized classification. A list of these hazardous substances for which harmonized classification and labelling has been established at the EU level can be found in Part 3 of Annex VI of the CLP regulation.

Under REACH, UVCBs are regulated based on the structural similarity of the constituents identified and can also be regulated as part of a different group. This means that certain components of these substances may be identified and registered as part of a different group. Annex XI Sect. 1.5 of REACH provides more information on the regulation of UVCBs [31, 70].

## Existing grouping legislation and impacts

### The Montreal Protocol on ozone-depleting substances

**Fig. 3:**
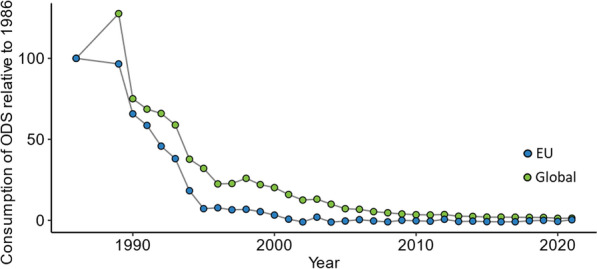
EU and global relative consumption of ODS since 1986 [74] showing the reduction of the consumption of ODS due to the Montreal Protocol

The Montreal Protocol regulates the manufacturing and consumption of ODS [64, 71]. This is a group of over 100 substances including chlorofluorocarbons (CFCs), methyl chloroform, hydrochlorofluorocarbons (HCFCs) and hydrobromofluorocarbons (HBFCs) that release chlorine and bromine into the stratosphere, damaging the ozone layer [64, 72]. This can lead to increased global warming, skin cancer and damage to marine ecosystems [73]. The Montreal Protocol is considered to have drastically decreased relative consumption (total of production and imports—total of exports and destroyed) of ODS in the EU (100–0.36%) and globally (100–1.35%) between 1986 and 2021 [71, 74], as shown in Fig. 3. This is an example of a property-based regulatory effort that has been successful [71, 75]. However, the Montreal Protocol has also been criticized for shifting the burden by transitioning from ODS to greenhouse gases and PMT/vPvM precursors, as discussed further below.

### The Stockholm convention on persistent organic pollutants

**Fig. 4:**
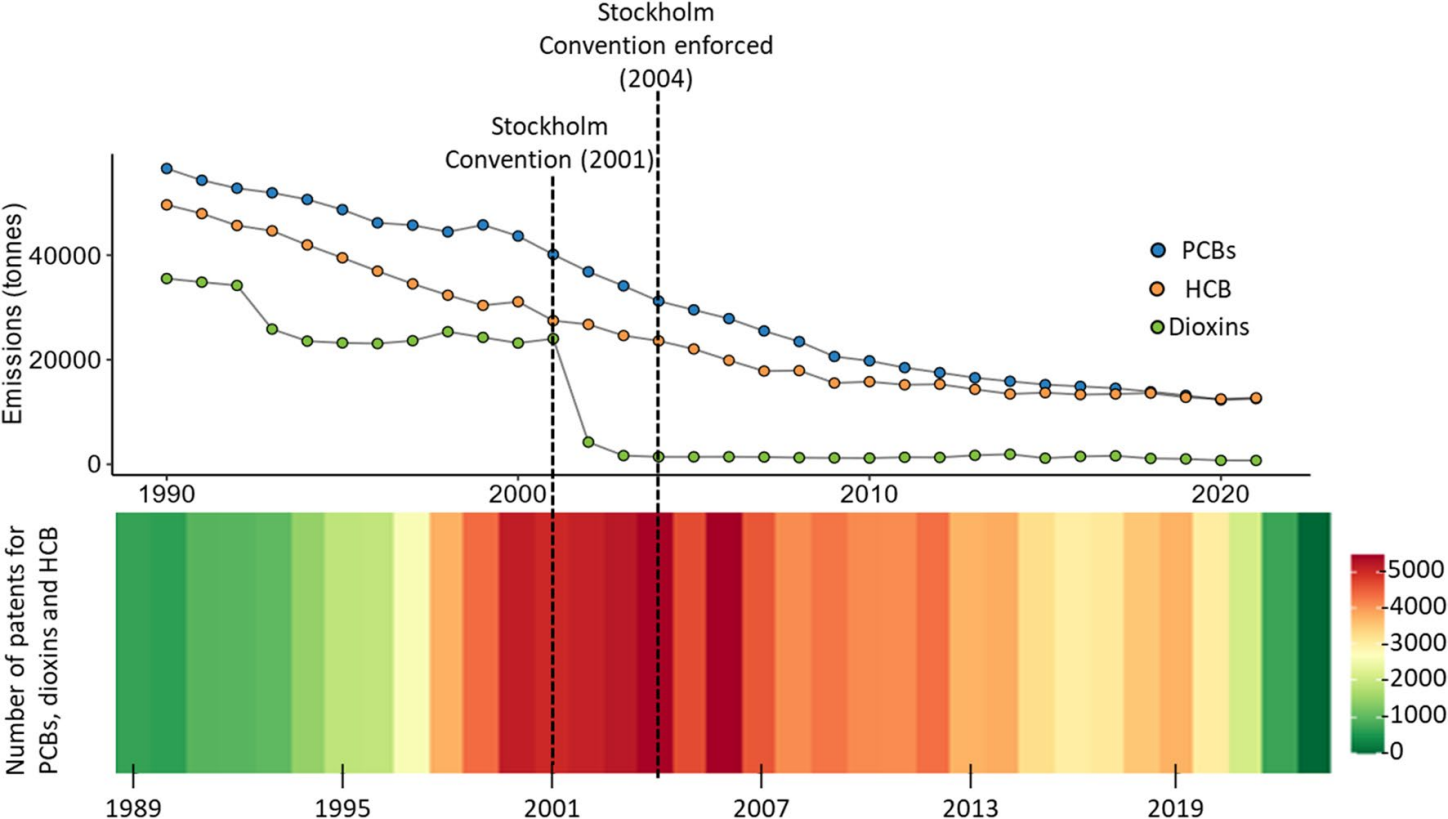
Top: Emission tonnage of PCB, dioxins and HCB from regulation (EU) No 277/2012, between 1990 and 2020 [80]. Bottom: The chemical stripes drawn using the chemical stripes package in R [81] show a decrease in the number of registered patents of PCB, dioxins and HCB in PubChem; the date range was chosen to match the emission data

The Stockholm Convention was signed in 2001 to protect humans and the environment from POPs [66]. An EU Legislation regulation (EC No 850/2004) was adopted later to implement the Convention in the EU [76, 77]. Initially, the Convention regulated 12 POPs or groups of POPs, such as aldrin, chlordane and dichlorodiphenyltrichloroethane (DDT). Over time, the list expanded to include 39 substances and substance groups as of January 2024, with the regulation covering the manufacturing, sales, use, and waste management of these chemicals, as well as unintentional releases in some cases [66, 78]. Structural similarity and the resulting similar compound properties played a major role in managing the grouping of substances under the Convention, for instance, polychlorinated biphenyls (PCBs), dioxins and DDT derivatives [79]. The Stockholm Convention has been successful in reducing the emission of regulated POPs since its enforcement. The emission levels of PCBs, for example, decreased by over 60% from 2001 to 2020, while dioxin levels also dropped by 50% from 2001 to 2020 (top of Fig. 4). This reduction is also noticeable as the number of registered patents for 17 dioxins, 12 PCBs and hexachlorobenzene (HCB) dropped since the Convention (bottom of Fig. 4).

### Substitution of regulated substances with PMT/vPvM substances

The concept of regrettable substitution first appeared in the literature in 2011 [82]. There are many examples of regrettable substitution, which refers to the practice of replacing a hazardous substance with a structurally similar substance for a specific use or function, that is also hazardous or potentially more hazardous, or that has not been tested broadly enough for different hazards and other than those of the substance being replaced [83]. One well-known example is Bisphenol A, an endocrine-disrupting chemical (EDC) that was first discovered in 1891 [84]. Due to its harmful effects, it has since been replaced by various other EDCs with similar structures, such as Bisphenol S and Bisphenol P. Currently, more than 30 bisphenols, including the ones mentioned above, are recommended for restriction by ECHA [85].

One of the reasons for the “drop-in” solution by companies after substance regulation (both as individual or as a group) is that structurally similar substances often have similar functions in the product being applied, and can be manufactured generally without requiring substantial changes in processes, infrastructure, or product testing [63]. Unfortunately, similar toxicological effects are likely to occur as well [26], and at other times “burden shifting” towards different hazards. A key example is the series of restrictions related to the Montreal Protocol that began with a global phase-out of CFCs [86, 87]. Later, hydrobromofluorocarbons (HBFCs) were introduced as replacements but were only briefly commercialized [88]. They were then replaced with hydrochlorofluorocarbons (HCFCs), which exhibited substantially less ozone-depleting potential, and exhibited the same commercial properties as CFCs [89]. HCFCs were eventually also included under the Montreal Protocol and were again replaced by chemicals with similar chemistry, hydrofluorocarbons (HFCs) [64]. Due to the removal of chlorine atoms from the molecules, HFCs no longer pose a threat to the stratospheric ozone layer [90], but they are still highly persistent and have high global warming potential. Therefore, in a subsequent amendment to the Montreal Protocol, the Kigali Amendment, HFCs themselves were listed [90, 91]. As a consequence of the Kigali amendment, the industry that manufactures fluorinated gases then sought to substitute HFCs with hydrofluoroolefins (HFOs), which have a short atmospheric lifetime of 6 days [92, 93]. However, these HFOs (almost) exclusively degrade to form trifluoroacetic acid (TFA), a vPvM substance that is ubiquitous in the environment, especially in drinking water systems, but the current understanding of its risk to human health and the environment is limited. While some research has argued that TFA has low health risks [94, 95], other studies have shown mild liver hypertrophy in rats, and eye and skin irritation [94, 96]. As the levels of TFA are rapidly increasing, some EU countries have proposed to ban HFOs as part of the broad PFAS restriction under REACH. In summary, although the Montreal Protocol is often considered the most successful multilateral environmental agreement, this has led to burden shifting over time from ODS to the production of persistent, mobile substances, such as TFA.

Similar to the Montreal Protocol, increased regulatory pressure on PBT/vPvB substances in Europe and the USA was seen as a driver for the chemical industry to produce more hydrophilic, and hence mobile, substances [97]. Individual fluoropolymer manufacturers have developed their own structurally similar per- and polyfluoroalkylether carboxylic acids (PFECAs) as replacements for PFOA, which was used as a processing aid in fluoropolymer production. However, as mentioned above, some of these replacements, including GenX, are similarly problematic despite being less bioaccumulative [98]. To date, many monitoring programs have seen an increase in these replacements in water resources and fish due to increasing use and emissions [99]. GenX and PFBS were identified as SVHCs under the EU REACH regulation in 2019 and were some of the first SVHCs that were considered an equivalent level of concern to PBT/vPvB substances [12]. Thus, the phasing out of some PBT/vPvB substances such as the long-chain PFASs has led to burden shifting towards PMT/vPvM substances such as PFECAs [100].

Existing regulation of substances as groups based on structural similarity and/or intrinsic properties has been successful, as demonstrated in the Montreal Protocol of ODSs and the Stockholm Convention of POPs. Meanwhile, regrettable substitution with increasing production of PMT/vPvM substances such as TFA and GenX has been noted as a side-effect. This shows a limitation of the grouping approach under the Montreal Protocol and the Stockholm Convention: limiting the scope of intrinsic properties during grouping may lead to burden shifting to other hazards. This calls for a grouping strategy that can prevent regrettable substitution and improve the efficiency and effectiveness of chemical regulatory processes.

## Grouping strategies

### Grouping based on structural similarity

Read-across in substance grouping is a strategy that relies on the use of important information obtained from tests conducted on a reference substance known as the “source” substance within a group to predict the properties of another substance in the group known as the “target” substance [101]. This method relies on justified similarities in structure, toxicokinetic, physicochemical and molecular properties, transformation process/endpoints and other similar data for interpolation and extrapolation of relevant information [102] and is recommended in Sect. 1.5 of Annex XI of the REACH regulation for groups of substances [70]. It can be applied from a single source substance to a single target substance (analogue approach) or from multiple source substances to multiple target substances within a group (category approach) [101, 103]. This method is commonly used to fill data gaps for chemical safety assessment in the regulatory process but has also been used to build grouping hypotheses for categories of chemicals [101, 102]. The Read-Across Assessment Framework (RAAF) by ECHA and the OECD guidance on the grouping of chemicals have been developed to guide systematic and consistent applications of read-across [104, 105].

Read-across is advantageous because it reduces the number of experimental tests needed during an assessment by using existing experimental data for the source substance(s) to predict the properties of the untested substance(s) [106]. If a clear hypothesis and justification are provided, read-across can be used efficiently to predict the hazard properties of target substances and fill data gaps in the regulatory process, thereby facilitating and speeding up assessment and regulatory decisions [107]. However, read-across requires an adequate justification for use, and appropriate documentation covering all assumptions and conclusions [36, 107]. This can be complex and may require a certain level of expertise for interpretation [108]. Despite its limitations, read-across can be applied to the grouping of PMT/vPvM substances.

For persistence in the environment, an important property is the readiness of the bonds to be broken down under ambient conditions, whether it is from radical reactions or through metabolic processes. For instance, the C–F bonds in PFAS (bond dissociation energy of 513.8 ± 10.0 kJ/mol), and aromatic–Cl bonds in PCBs or dioxins (394.9 ± 13.4 kJ/mol), are difficult to break down in the environment, making groups rich in such bonds likely to be persistent [109]. Compounds with molecules containing high bond dissociation energies can be flagged for persistence assessment and subsequent inclusion as PMT/vPvM if each of the M and/or T criteria is met. Biodegradability models can be used to quickly provide relevant information about the persistence of many substances or the presence of many persistent substructures, pending further investigation and confirmation by experimental studies [110]. However, the applicability domain of available models is dependent on their training data, resulting in unreliable predictions for compound classes that may not be covered yet [110].

For mobility, the chemical substructures that are associated with low *K*_OC_ values are those that are highly polar or ionic, as this favours their water solubility over sorption to soil organic carbon [24, 111]. A 2022 review found that most ionic compounds with measured log *K*_OC_ values have log *K*_OC_ values < 4.0 [24]. Thus, the presence of many hydrophilic substituents may be a predictor of mobility, and conversely, chemicals with largely hydrophobic substructures are unlikely to be mobile in the environment. However, an important limitation of mobility is size. Extremely large molecules that are highly polar and ionic may not be mobile if they have sufficient hydrophobic substructures to decrease their solubility, or (as in the case of water-soluble polymers) can aggregate for charge neutrality and therefore lose/reduce their mobility as an aggregate [112]. Quantitative structure–activity relationship (QSAR) models are available to predict the mobility of certain substances and can be applied effectively (within their respective applicability domains) to classify the mobility of substances.

The toxicity of a substance is related to its chemical structure and determines the type of health effects it induces in the biological systems [113]. The interaction of a chemical with a biological system is determined by the functional groups, stereochemistry and other molecular features [114]. A change in the structure can lead to changes in toxicity, depending on how this change affects the toxicophore—the structural portion associated with the toxicity of the chemical [115]. The descriptors used for toxicity predictions vary between models, some models consider only 2D descriptors, while others use a mix of 2D and 3D descriptors. In recent years, some models have also attempted to predict toxicity through graph neural networks which take the position of the different functional groups into consideration [116, 117]. Toxicity is also related to the absorption, distribution, metabolism and excretion ability of the body, which depends on the chemical structure of the substance. Increased hydrophobicity leads to increased absorption and increased potential toxic effects (due to potential bioaccumulation). A concern with PMT/vPvM substances is chronic exposure via water consumption, which can lead to elevated concentrations in humans and diverse biota [12, 118].

Considering the chemical features that make these substances P, M and T, and the provided criteria for classification, PMT/vPvM substances can be identified and grouped accordingly. Read-across can be applied to identify PMT substances on the basis that they have similar (sub)structures, similar properties and available toxicological data. This can be done through a fragment-based approach, which relies on the identification of small similar fragments or functional groups with similar properties [119]. Substance grouping approaches take the fragment models one step further. Substances can have similar properties due to the similarity in structures—this includes functional groups, common precursors or reaction products, and a constant pattern in the changing of the potency of the properties across the group [31, 104]. The substance grouping approach is used in read-across techniques and alternative assessments [104]. This has been supported by the use of various models that predict physical–chemical property information such as the KOCWIN model [120]. This is a fragment-based model that relates substructures and mobility (*K*_OC_) based on appropriate training data [120]. The mobility (*K*_OC_) of new chemicals can be predicted using the established relationship (when they are within the applicability domain). Similarly, the ReadyBiodegradable model in OPERA [36] and CERAPP and CoMPARA models [116, 121]can be used to predict the biodegradability and toxicity, respectively, of many new PMT/vPvM substances.

### Grouping based on retained moieties from transformation reactions

Both biotic and abiotic transformations generally result in transformation products (TPs) with significantly higher mobility (lower log *K*_OW_) than their parent compounds or precursors, making them more mobile in the environment. This seems intuitive as one of the "goals'' of metabolism and wastewater treatment processes is to increase the polarity of the compounds. For metabolic processes, this allows the compounds to be expelled from the body with the urine. However, some persistent substructures may be retained during the transformation resulting in similar toxic properties between the parent compound and TPs. For example, decabromodiphenyl ethane forms 6 metabolites that were found to be carcinogenic like the parent compound [122]. In other cases, even small structural changes can result in large differences in toxicity such as for bis(pentabromophenyl) ether, which has low thyroid binding affinity itself but can form the TP 2,3,5,6-tetrabromo-4-(2,3,4,5,6-pentabromophenoxy)phenol with a strong thyroid binding affinity [122]. Therefore, it is of interest not only to identify parent compounds that may be PMT/vPvM, but also compounds that can form PMT/vPvM TPs, along with identifying the common persistent and mobile moieties that were retained and not readily metabolized. Such an approach would be consistent with the approach to substance grouping in REACH of identifying “common breakdown products via physical and biological processes, which result in structurally similar chemicals” (REACH Annex XI, Sect. 1.5).

One way to do this identification is by using known reactions, such as those contained within the “transformations” section in PubChem, which links parent compounds to TPs with basic reaction information [123]. Of all the substances on the five suspect lists mentioned above, perfluorooctanoic acid (PFOA) has the highest number of recorded parent compounds (22) in the PubChem transformations section (from version 0.1.6 of the data set archived on Zenodo) [124]. This is also one of several PMT/vPvM TPs, where parent compounds are included in the legislation as part of the substance group. Specifically, the Stockholm Convention restricts the use of PFOA-related compounds, which include compounds that form PFOA after degradation [78]. TFA is also a known TP of several different compounds containing a CF_3_–moiety. Since there are over 5 million compounds containing a CF_3_–moiety included in PubChem [125], many of which are also industrially relevant, there are myriad potential sources of TFA.

Triazoles are another example of moieties preserved from parent compounds to TPs. Out of the triazole compounds in PubChem, 62 have recorded TPs (12 Jan 2024) [124]. This corresponds to 233 unique reactions, out of which the triazole moiety is retained in 88% of the cases, showing its high stability. While usually not acutely toxic in low doses, triazoles may cause several severe chronic toxic effects, such as endocrine disruption and neurotoxicity [126]. Triazole substances such as benzotriazoles are present in the environment at high levels [126]. Thus, the retention of this functional group and other PMT/vPvM moieties may be concerning from an exposure point of view, supporting the idea that grouping based on retained moieties or TPs is relevant to obtain comprehensive and inclusive groups for PMT/vPvM substances.

Another example of how substances can be regulated based on their precursors are the aromatic amines registered under REACH some of which can also be found on PMT/vPvM suspect lists, such as 4,4′-methylenedianiline, 4,4′-oxydianiline, 4-chloroaniline, 3,3′-dichlorobenzidine, 4,4′-methylenebis(2-chloroaniline), 4,4′-methylenedianiline, and 2-methoxy-5-methylaniline [20, 31, 50]. However, there are several other aromatic amines that have multiple known precursors listed in the PubChem transformations library (e.g., 4-aminophenol and aniline) that are also listed as PMT by the UBAPMT suspect list. As such, regulating aromatic amines and precursor substances as a group based on PMT/vPvM substances warrants further investigation and consideration.

## Cheminformatics challenges in grouping PMT/vPvM substances

The strategies mentioned above regarding grouping can be matched to substance regulation in various ways. First, substances falling within a PMT/vPvM group with little known data could be flagged for follow-up to see if persistence and mobility measurements have been conducted in literature, and if read-across methods (based on patterns with other substances) could be used to fill data gaps. This could help prioritise filling data gaps and would help develop read-across approaches for further substances within the group. Second, if any other substance with a similar structure exists in the group and is hazardous (source substance), this could be seen as a reason to investigate for similar toxicological hazards for this substance (target substance). If several members within a group are shown to be PMT/vPvM substances, then a precautionary approach would be to assume all group members with no assessments are similarly hazardous, until there is sufficient scientific data to show otherwise. An overview of an assessment procedure to identify PMT/vPvM substances has also been presented by Neumann and Schliebner, suggesting to first assess compounds for persistence followed by mobility and toxicity for both precursors and transformation products [47].

Estimating the number of individuals and groups of chemicals that fit the PMT/vPvM classification is a challenging task. Most substances lack readily available persistence, mobility and toxicity data. Due to the limited data availability, the prediction models of these properties are also limited in their accuracy and applicability domains. However, some data and models are available as a starting point, especially for log *K*_OW_. For example, both XlogP and the newer XlogP3 [127, 128] and the KOWWIN [129] module in EPI Suite [130] use a multivariate regression approach to *K*_OW_ prediction. While *K*_OC_ data are more limited than *K*_OW_ data, there are also several models which attempt to predict *K*_OC_, including KOCWIN from EPI Suite [37] and the *K*_OC_ module of OPERA [36], which both utilise the training data from the PHYSPROP database [36, 37]. For persistence, OPERA also contains three modules for predicting biodegradability (BiodegHL, ReadyBiodegradable and Km) as well as one module predicting rate constants for gas-phase reactions with hydroxyl-radicals, though with more limited applicability domains compared to the *K*_OC_ module [36]. The prediction of toxicity is even more challenging due to the limited availability of training data. However, models already exist for several toxicological endpoint predictions, such as endocrine disruption, mutagenicity and developmental toxicity for many possible structures [131, 132]. For example, the CERAPP and CoMPARA models in OPERA can be used to predict estrogen and androgen receptor interactions [116, 121].

An example of how a combination of experimental and predicted property data has been used to classify compounds as PMT/vPvM is the study by Arp and Hale (2022), which focused on the compounds in the REACH inventory [24]. Their study concluded that about 1.9% of substances registered in REACH and 24% of REACH-registered substances that were detected in drinking water sources would be classified as PMT. However, over 40% of the inventory could not be assessed due to data gaps. This illustrates the importance of acquiring experimental data for a wider range of compounds.

**Fig. 5:**
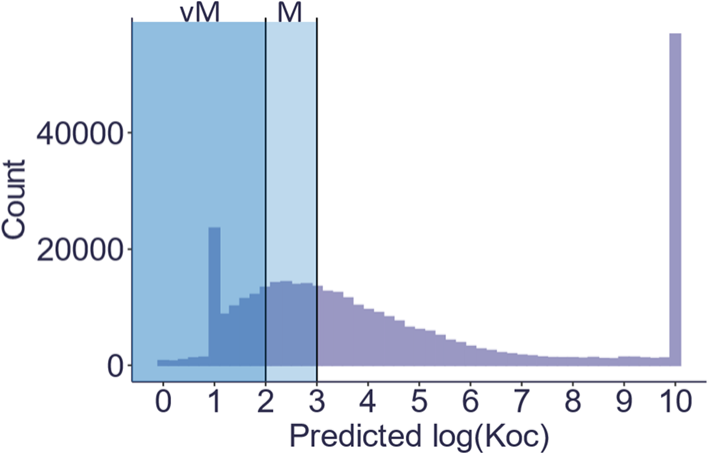
Predicted log K_OC_ via KOCWIN values for PubChemLite (346,133 compounds total) [38, 133]. Some K_OC_ predictions fall outside the applicability domain of the model. The “count” is the number of compounds inside each bin. Bin widths are 0.2

To further illustrate how predicted properties can be used for assessing PMT compounds, the PubChemLite for Exposomics [133] data set was used to give a preliminary estimate of the number of potential environmental contaminants that are predicted to meet the CLP definition of mobility. PubChemLite is a subset of ~ 350,000 compounds from PubChem with environmentally relevant annotation content [133]. In total, *K*_OC_ values were predicted for 346,133 compounds (96.0% of PubChemLite) using the molecular connectivity index (MCI) model from KOCWIN [37, 120]. The results of the log *K*_OC_ distribution are shown in Fig. 5. Based on these predicted values log *K*_OC_, 147,930 compounds (41%) would be considered mobile and 78,107 compounds (21%) would be considered very mobile. The MCI model assigns values of 1 and 0 for chemicals when *K*_OC_ predictions have been overcorrected. The large peak at log *K*_OC_ 10 is an upper bound for these predictions within the MCI model output. Note that the results of this analysis will differ if the *K*_OW_-based model in KOWWIN is used.

To investigate this concept in more detail on slightly smaller subsets, four compound classes: triazines, aromatic amines, PFAS and triazoles were selected based on the five suspect lists mentioned previously (UBAPMT, EAWAGPMT, UFZHSFPMT, PMTPFAS and ZEROPMBOX1) [20, 49–52]. The substructure search function of PubChem was then used to estimate the number of compounds that belong to these compound classes. An overview of these search queries can be found in Table 1. As can be seen in the first three rows, relatively unspecific substructures can generate very large query results. For the 1,2,3-triazole substructure over 1 million substances were found, which corresponded to a search of only 87% of the database as the search results are capped at 1 million substances. Similarly, the aromatic amines (with aniline as the searched substructure) resulted in over 1 million substances with only 2% of the database searched. The 1,3,5-triazine query resulted in 718,119 substances. The compounds classified within the OECD definition of PFAS in the PubChem PFAS tree were used to source PFAS, which contained 6,604,017 compounds at the time the queries were performed (Dec. 17, 2023) [125]. It is unlikely that all compounds containing these substructures would meet the PMT/vPvM criteria outlined in the CLP. For example, only 250,873 of the 718,199 triazines meet the CLP criteria for mobility based on the predicted log *K*_OW_ from XlogP3. As such the substructures used when discussing PMT/vPvM compound grouping for regulation purposes should be more specific.

**Fig. 6:**
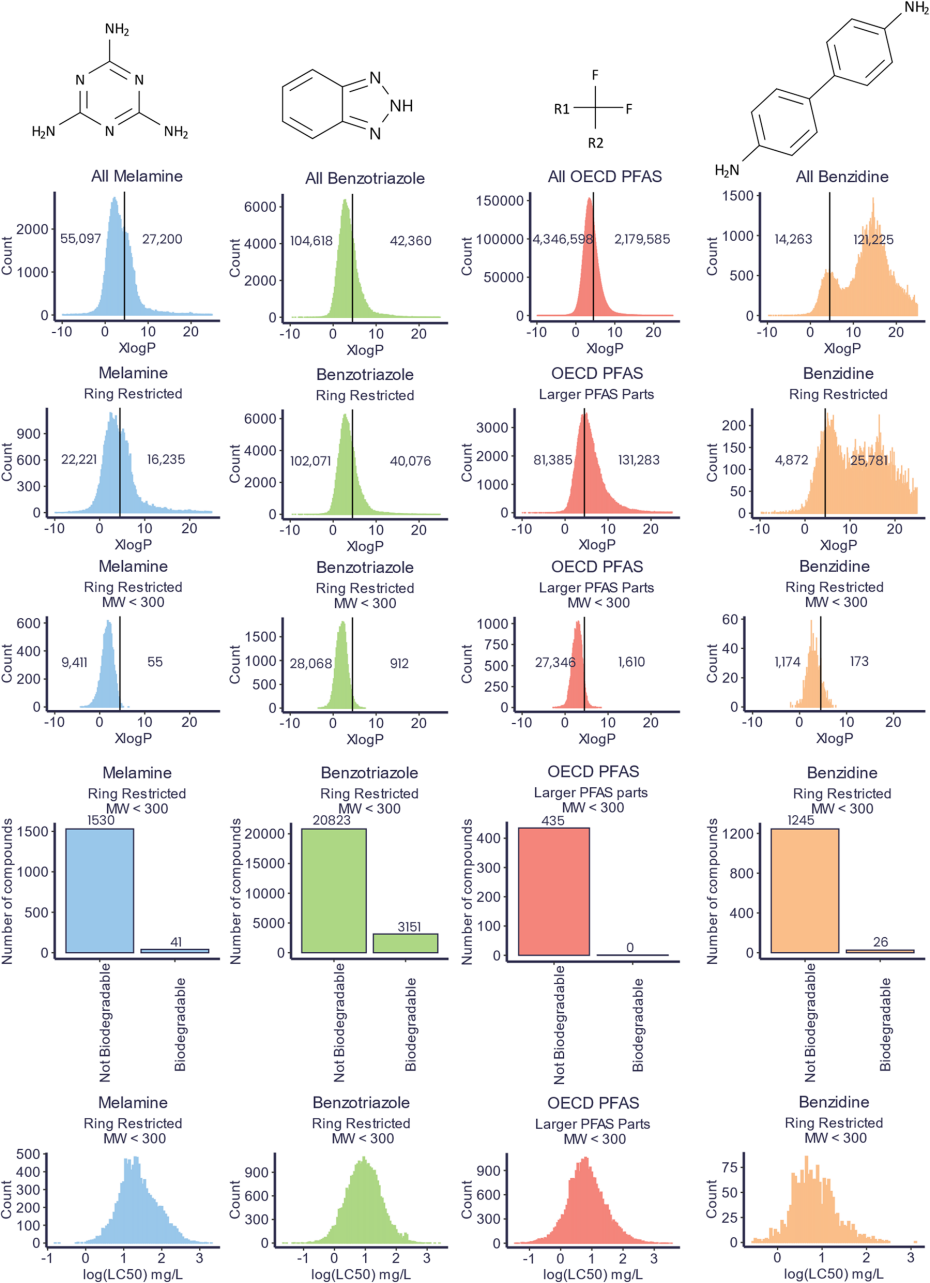
Distribution of PubChem XlogP3 values for four searches with varying restrictions (top three rows), plus predicted biodegradability from the OPERA ReadyBiodegradable model and LC_50_ values (in mg/L) predicted from MS2Tox for the most restricted search queries (last two rows). Search settings and total numbers are given in Table 1. It should be noted that MS2Tox is used for illustration purposes only, and does not reflect the CLP toxicity criteria in Fig. 1

More specific substructure queries were used to interrogate these results further according to the PMT/vPvM criteria, shown in the remaining rows of Table 1 and Fig. 6, using the structures of melamine, benzotriazole and benzidine as well as the “larger PFAS parts” definition (contains –CF_2_CF_2–_) from the PubChem PFAS tree [125]. The search was then progressively restricted to exclude the searched substructures from being part of larger ring systems and substances with molecular weights greater than 300 g/mol. The results of this search were also used to perform persistence and toxicity prediction as discussed above. The top two rows of Fig. 6 show these substructure query results. Like their less specific triazine counterpart, they seem too broad to capture only PMT/vPvM substances. However, when restricting the search to only compounds below 300 g/mol, between 87% and 99% of the compounds would meet the CLP definition of mobility. The most restricted PubChem search results were also used for biodegradability and LC_50_ predictions using the ReadyBiodegradable model from OPERA and MS2Tox (compounds which fell outside the applicability domain of the ReadyBiodegradable model were excluded in the biodegradability results; this information is not given by MS2Tox). As can be seen in Fig. 6, almost all compounds inside the applicability domain were classified as non-biodegradable. This indicates that in addition to their mobility, most compounds within these classes are potentially persistent. Based on the EU CLP definition of toxicity, an EC_10_ less than 0.01 mg/L is toxic for marine or freshwater organisms. Some of the predicted LC_50_ toxicity values (the lethal concentration at 50% of the population) shown in Fig. 6 go below 0.1 mg/L. As toxicity is usually observed before lethality, this indicates that some of the compounds may be toxic below 0.01 mg/L. In addition, it is important to note that this is not the only toxicity criterion used in the CLP, for example, triazole compounds have been shown to have endocrine-disrupting properties by, e.g., Wang et al. [134], and thus, it is likely that at least a fraction of these compounds would be classified as PMT.

**Table 1:** Overview of the PubChem substructure searches (queries performed on 19/12/2023)

Substructure	Substructure SMARTS	Extra settings*None*	Number of structures
*1,3,5-Triazine*	C1=NC=NC=N1	*None*	*718,134*
*Aromatic amine*	C1=CC=C(C=C1)N	*None*	> *1 M (2% of DB)*
*1,2,3-Triazole*	C1=NNN=C1	*None*	> *1 M (87% of DB)*
Melamine	C1(= NC(= NC(=N1)N)N)N	None	96,660
Melamine	C1(= NC(= NC(=N1)N)N)N	Ring	47,519
Melamine	C1(= NC(= NC(=N1)N)N)N	Ring, MW < 300	10,528
Benzotriazole	C1=CC2=NNN=C2C=C1	None	165,361
Benzotriazole	C1=CC2=NNN=C2C=C1	Ring	158,766
Benzotriazole	C1=CC2=NNN=C2C=C1	Ring, MW < 300	30,945
Benzidine	C1=CC(= CC=C1C2=CC=C(C=C2)N)N	None	208,934
Benzidine	C1=CC(= CC=C1C2=CC=C(C=C2)N)N	Ring	48,123
Benzidine	C1=CC(= CC=C1C2=CC=C(C=C2)N)N	Ring, MW < 300	1,618
OECD PFAS	PFAS tree—OECD PFAS definition	None	6,604,017
OECD PFAS	PFAS tree—OECD PFAS definition	Larger PFAS Parts	222,174
OECD PFAS	PFAS tree—OECD PFAS definition	Larger PFAS Parts, MW < 300	29,521

Default settings for all queries were (1) single or double bonds match aromatic bonds, (2) chain bonds in the query may match rings in hits and (3) remove any explicit hydrogens before searching. Extra settings “Ring” (rings may not be embedded in a larger system) and the filter “MW < 300” (Molecular weight < 300 g/mol) are indicated in the respective column. Query URLs are embedded into the substructure column. The first three rows in italics are generic queries, the latter rows are the queries used to generate Fig. 6. The OECD PFAS results were obtained via the PubChem classification browser. DB = database, values in brackets indicate the percentage of the database searched (queries are capped at 1 M results).

## Perspectives for grouping strategies

### Challenges for assessing and managing PMT/vPvM substances

Based on the criteria for persistence, mobility and toxicity, many chemicals (as shown from the prediction results above) may be PMT/vPvM substances, warranting further assessments and management (see Table 1). One of the major challenges of dealing with PMT/vPvM substances is the lack of high-quality data. As shown above, there is a growing need for computational modelling to complement experimental approaches as the number of substances to be tested increases, yet efficient computational methods rely on high quality and sufficient experimental data availability, which is a major limiting factor. Structurally diverse substances with multiple functional groups and stereochemistry can lead to unexpected behaviour, posing a critical challenge [135]. However, developing high-throughput screening (HTS) methods for toxicity testing may be difficult for these diverse substances and toxicological endpoints, which produce large amounts of data, and require expert knowledge to interpret and manage [136].

Identification, monitoring and removal are other challenges to managing PMT/vPvM substances. Targeted identification and monitoring approaches will not be sufficient to detect all PMT/vPvM substances; hence, it is crucial to ensure wider availability of analytical methods and reference standards to properly identify, quantify and assess potential PMT/vPvM substances. Targeted analysis could be complemented with non-targeted analytical approaches. Non-target analysis can be a first step in identifying unknown and emerging PMT/vPvM substances and their TPs in environmental and biological matrices. It can also be used for the monitoring of known PMT/vPvM substances, yet regulatory acceptance of non-targeted monitoring is still lacking [137]. Moreover, the lack of data on persistence, mobility, toxicity, TPs, complex mixtures and appropriate suspect lists for broader suspect screening poses identification and monitoring challenges [22].

### Prioritization strategies for testing and assessment

Considering many potential PMT/vPvM substances, and the possible challenges outlined, it is necessary to develop other strategies that could be used in combination with the proposed grouping strategies above to prioritize chemicals for assessing their PMT/vPvM properties. Exposure and emission information could serve in the prioritization of chemicals for testing. Utilising exposure information such as mode of exposure (chemicals in food or drinking water) [138, 139] or Occupational Exposure Banding strategy for categorization of airborne substances lacking defined limits [140], could help identify chemicals with high exposure potential. In addition, the hazard level of the toxicity can be used as a criterion, where specific persistent and mobile substances with high toxicity can be targeted for prioritization.

The use of in vitro, in vivo, and in silico approaches can aid the grouping and prioritization of chemicals for testing or assessments by regulatory bodies. This can be done by generating grouping hypotheses and justification for inclusion or exclusion criteria for substances in groups [140]. In particular, novel approaches may generate huge amounts of toxicological and high-throughput “omics” data including metabolomics, transcriptomics, and exposomics to support the validation and establishment of grouping hypotheses needed by regulatory authorities [141]. An example of a grouping hypothesis is the mode of action (MOA) hypothesis, which states that all chemicals that share a common mode of action are candidates for grouping [142]. In addition, concepts such as adverse outcome pathways and toxicity pathways can be translated into prioritization hypotheses that can target specific substances, hence advancing prioritization for hazard assessment [143, 144]. These can facilitate regulator efforts to restrict hazardous substances.

Measurable biological and physiological effects of chemical exposure, known as effect biomarkers, can be used to group PMT/vPvM substances according to their toxicological profiles. If two substances induce similar biomarker responses indicative of for instance genotoxicity, or endocrine disruption, they can be grouped. This approach can also help to identify common pathways of toxicity, adverse outcome pathways (AOPs), or mechanisms of toxicity. By grouping substances with similar toxic profiles as PMT/vPvM substances, effect biomarkers can aid in characterizing cumulative risks posed by mixtures and complex chemical substances. This proactive approach focuses on biological effects rather than relying solely on the structural and chemical properties of PMT/vPvM substances [145, 146]

It is crucial to recognize that the effectiveness of strategies used for prioritizing substances largely depends on the availability of relevant data, including hazard and exposure data. To improve the availability and quality of such data, it is necessary to foster collective efforts, such as high-quality data generation, community-level data collection, Open Science, FAIR data, and enhanced data sharing policies. When substances are identified by read-across and experimental evidence as substances that meet the PMT/vPvM criteria, then a hazard classification would be required. The data collected through these efforts can then be used to enhance the prioritization, identification, and regulation of PMT/vPvM substances. To manage PMT/vPvM substances effectively, manufacturers can be encouraged to submit a plan to prevent emissions and remove these substances from wastewater. This approach can help improve the overall management of PMT/vPvM substances. As a precaution, the principle of "as low as reasonably achievable" (ALARA) can be applied to minimize exposure levels and reduce the risk of harm to PMT/vPvM substances [147].

## Conclusion

To achieve the EU’s zero pollution ambition of a non-toxic environment by 2050, regulating the production, use and disposal of PMT/vPvM substances is necessary. As shown in Table 1, scaling PMT/vPvM criteria to big substance collections reveals that there are potentially thousands of PMT/vPvM substances that could cause harm to human health and the environment, especially concerning water quality and drinking water treatment.

The Montreal Protocol and the Stockholm Convention have demonstrated that grouping substances can be an effective strategy to expedite the elimination of the production, use, and emission of toxic substances, such as ozone-depleting substances (ODSs) and persistent organic pollutants (POPs). This approach can also accelerate the identification and regulatory processes for substances that lack hazard information. Grouping can prevent the introduction of new hazardous substances into the global market. However, it is important to ensure that grouping is done in a way that is feasible and promotes the use of safer and more sustainable alternatives. Otherwise, it could result in the production of regrettable substitutes, as was the case with ODSs and POPs, which led to the creation of some PMT/vPvM substances.

Read-across based on structural or substructural similarity is one of the strategies that could be used to group PMT/vPvM substances, which relies on the idea that substances with similar structures have similar properties. Commonly retained moieties from transformation reactions could also be a grouping strategy for PMT/vPvM substances. Substances that are structurally similar to PMT/vPvM according to read-across, or form persistent, mobile TPs could be flagged for subsequent assessment and/or regulatory actions.

Cheminformatics may be used for substance grouping based on predictive models for properties, such as biodegradability, mobility, and toxicity. PubChemLite predictions suggest that 41% of potentially environmentally relevant compounds would be considered mobile (147,930 compounds) and 21% would be considered very mobile (78,107 compounds). Certain compound classes, such as triazines, aromatic amines, triazoles and PFAS, are likely to be persistent, non-biodegradable, and toxic. As shown in Table 1, the numbers are high and restricting these compounds as a group would be challenging; however, prioritizing members of these large groups for property testing is warranted as they contain a substructure associated with a PMT/vPvM substance group. Additional strategies are needed to prioritize some substances for regulation, such as mapping the uses of these chemicals and exposure which requires more data availability following FAIR principles.

Some strategies proposed for the prioritization of substances for testing or assessment of PMT/vPvM include (i) better understanding and the use of exposure/ emissions information, such as Occupational Exposure Banding and environmental exposure to the PMT/vPvM substances, and (ii) the use of in vitro, in vivo and in silico techniques to generate relevant toxicological data that will support identification, prioritization and regulation of PMT/vPvM substances. These strategies in combination with substance grouping could result in substituting PMT/vPvM substances with safer alternatives.

## Data Availability

The data sets analysed during the current study are available in PubChem, the NORMAN Suspect List Exchange (NORMAN-SLE—https://www.norman-network.com/nds/SLE/) and on Zenodo under the following URLs: S36 UBAPMT (10.5281/zenodo.6482414), S82 EAWAGPMT (10.5281/zenodo.5500132), S84 UFZHSFPMT (10.5281/zenodo.5535288), S90 ZeroPMBox1 (10.5281/zenodo.5854252), S111 PMTPFAS (10.5281/zenodo.8417075), PubChemLite for Exposomics (Version 1.27.0, Oct. 27th 2023, 10.5281/zenodo.10126889, PubChem Transformations Data set (Version 0.1.6, Jul. 5th, 2023, 10.5281/zenodo.8117741) and the PubChem PFAS Tree (https://pubchem.ncbi.nlm.nih.gov/classification/#hid=120).

## Abbreviations

CAS: Chemical Abstracts Service
CFCs: Chlorofluorocarbons
CID: PubChem Compound Identifier
CLP: Chemicals, labelling and packaging
DDT: Dichlorodiphenyltrichloroethane
*D*_OW_: PH-dependent octanol–water partition coefficient
EC: European Commission
*EC*_*x*_: Effect concentration at X percentage
ECHA: European Chemical Agency
EDC: Endocrine-disrupting chemical
EU: European Union
HBFCs: Hydrobromofluorocarbons
HCB: Hexachlorobenzene
HCFCs: Hydrochlorofluorocarbons
HFCs: Hydrofluorocarbons
HFOs: Hydrofluoroolefins
HTS: High-Throughput Screening
IUPAC: International Union of Pure and Applied Chemistry
KEMI: Swedish Chemical Agency
*K*_OC_: Organic–carbon–water partition coefficient
*K*_OW_: Octanol–water partition coefficient at pH = 7
log *P*: Logarithm of *K*_OW_
M: Mobility
MCI: Molecular Connectivity Index
MOA: Mode of action
NOEC: No observable effect concentration
NORMAN-SLE: NORMAN suspect list exchange
ODS: Ozone-depleting substances
P: Persistence
PBT: Persistent, bioaccumulative and toxic
PCBs: Polychlorinated biphenyls
PFAS: Per- and poly-fluoroalkyl substances
PFBS: Perfluorobutane sulfonic acid
PFECAs: Per- and polyfluoroalkylether carboxylic acids
PFOA: Perfluorooctanoic acid
PMT: Persistent, mobile and toxic
PMT/vPvM: Persistent, mobile and toxic or very persistent and very mobile
POPs: Persistent organic pollutants
QSAR: Quantitative structure–activity relationship
RAAF: Read-across assessment framework
REACH: Registration, evaluation, authorisation and restriction of chemicals
SCHEER: Scientific Committee on Health, Environmental and Emerging Risks
SVHCs: Substances of very high concern
T: Toxicity
TFA: Trifluoroacetic acid
TPs: Transformation products
UBA: Umweltbundesamt (German Environmental Agency)
UVCBs: Substances of unknown or variable composition, complex reaction products or biological origin
vM: Very mobile
vP: Very persistent
vPvB: Very persistent and very bioaccumulative
vPvM: Very persistent and very mobile
